# Enhancing Activation Energy Predictions under Data Constraints Using Graph Neural Networks

**DOI:** 10.1021/acs.jcim.4c02319

**Published:** 2025-01-25

**Authors:** Han-Chung Chang, Ming-Hsuan Tsai, Yi-Pei Li

**Affiliations:** † Department of Chemical Engineering, National Taiwan University, No. 1, Section 4, Roosevelt Road, Taipei 10617, Taiwan; ‡ Taiwan International Graduate Program on Sustainable Chemical Science and Technology (TIGP-SCST), No. 128, Section 2, Academia Road, Taipei 11529, Taiwan

## Abstract

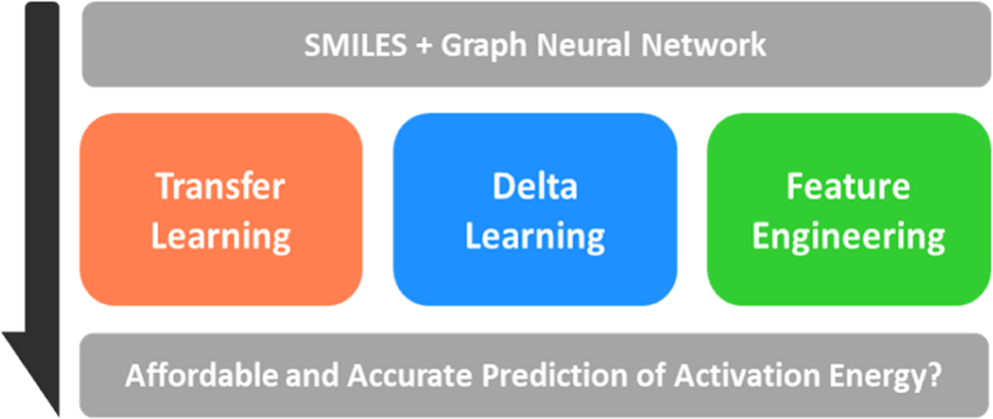

Accurately predicting activation energies is crucial for understanding chemical reactions and modeling complex reaction systems. However, the high computational cost of quantum chemistry methods often limits the feasibility of large-scale studies, leading to a scarcity of high-quality activation energy data. In this work, we explore and compare three innovative approaches (transfer learning, delta learning, and feature engineering) to enhance the accuracy of activation energy predictions using graph neural networks, specifically focusing on methods that incorporate low-cost, low-level computational data. Using the Chemprop model, we systematically evaluated how these methods leverage data from semiempirical quantum mechanics (SQM) calculations to improve predictions. Delta learning, which adjusts low-level SQM activation energies to align with high-level CCSD(T)-F12a targets, emerged as the most effective method, achieving high accuracy with substantially reduced data requirements. Notably, delta learning trained with just 20–30% of high-level data matched or exceeded the performance of other methods trained with full data sets, making it advantageous in data-scarce scenarios. However, its reliance on transition state searches imposes significant computational demands during model application. Transfer learning, which pretrains models on large data sets of low-level data, provided mixed results, particularly when there was a mismatch in the reaction distributions between the training and target data sets. Feature engineering, which involves adding computed molecular properties as input features, showed modest gains, particularly in thermodynamic properties. Our study highlights the trade-offs between accuracy and computational demand in selecting the best approach for enhancing activation energy predictions. These insights provide valuable guidelines for researchers aiming to apply machine learning in chemical reaction engineering, helping to balance accuracy with resource constraints.

## 1 Introduction

Advancements in ab initio quantum chemistry methods have significantly expanded the ability of chemists to perform complex reaction simulations, leading to considerable progress in various fields such as material design and discovery,^1,2^ photovoltaic materials,^3,4^ and the elucidation of catalytic mechanisms with enhanced insight into chemical reactions.^5−8^ These sophisticated computational techniques enable the calculation of activation energies, a crucial parameter for the quantitative understanding of challenging chemical problems and an essential kinetic factor in reaction simulations. However, due to the high computational costs associated with high-level quantum chemistry methods, large-scale theoretical studies are often constrained. Consequently, initial screening typically relies on empirical methods to rapidly estimate activation energies, thereby narrowing the focus to the most critical reaction pathways that warrant computationally intensive quantum chemistry calculations.

Numerous models have been developed to estimate kinetic parameters,^9−14^ each offering unique approaches and advantages. For instance, the reaction mechanism generator^15^ (RMG) software employs a hierarchical decision tree to match a given reaction with similar ones from its database, extracting and averaging their kinetic parameters for estimation. This method provides a convenient and interpretable means to quickly estimate the kinetic parameters, particularly for well-characterized reaction types. The quantitative structure–activity relationship (QSAR) model has also been utilized to explore the relationship between kinetic properties and the physicochemical descriptors of reactants and products.^16,17^ For instance, van Gerwen et al. extend quantum machine learning (ML) to predict reaction properties by introducing reaction representations based on atomic charges and coordinates;^18^ Hoonakker et al. introduced the condensed graph of reaction (CGR) encoding method, which combines reactants and products into a single pseudomolecule, enabling the use of standard cheminformatics methods for featurizing reactions and predicting their kinetic parameters.^19^

With the rise of deep learning, artificial neural networks (ANNs) have been applied to predict kinetic parameters,^20,21^ with the multilayer perceptron (MLP) approach demonstrating success in various reaction systems, including aqueous OH reactions,^22^ OH radical reactions,^23^ and tropospheric ozone degradation.^24^ However, these methods typically rely on user-defined molecular descriptors, limiting their generalizability across different reaction types. More advanced deep learning architectures, such as those based on graph representations, SMILES strings, and three-dimensional (3D) molecular structures, have been developed to automatically learn implicit information from molecular connectivity,^25−46^ often outperforming traditional ANN models. For example, Grambow et al. expanded the applicability of kinetic predictions using a template-free graph-based model, employing dual directed-message passing neural network (D-MPNN) to encode reactant and product graphs for activation energy prediction.^47^

Despite the considerable success of machine learning (ML) methods in predicting molecular properties,^48,49^ there remain significant challenges, particularly concerning data scarcity. High-quality kinetic parameters are often limited, necessitating the development of techniques that enhance model performance without relying on extensive data sets. One such approach involves incorporating additional data computed using lower-level theories to augment the training set. However, identifying which data to compute and how to effectively integrate them into models remains an area of active research. Feature engineering is one strategy for addressing these challenges. By calculating properties of reactants and products relevant to their kinetic behavior using lower-level methods, such as semiempirical quantum mechanical (SQM) techniques, these features can be incorporated into the model to improve performance.^50^ For example, Marques et al. demonstrated accuracy improvements by integrating electronic structure information,^51^ while Choi et al. utilized molecular structure and thermodynamic properties to predict gas-phase reaction activation energies.^52^ Another promising approach is transfer learning, where a model is pretrained on a large data set before being fine-tuned on a smaller, target-specific data set to improve model performances. In the context of activation energy predictions, models can be initially trained on extensive data sets derived from low-level calculations and subsequently fine-tuned using a smaller set of high-quality data, thereby reducing the computational cost of data generation. Previous studies, such as those by Spiekermann et al. and Espley et al., have explored the benefits of transferring models trained at lower theoretical levels to higher-level data, with notable improvements in prediction performance.^53,54^ A relatively novel approach, delta learning, has also emerged as a viable strategy.^55,56^ Delta learning involves predicting the difference between low- and high-level computed data, allowing the model to learn corrections to low-level computations. This approach has demonstrated promising results, as seen in studies where delta learning was applied to graph neural network (GNN) models to predict high-level data from lower-level calculations.^57−62^

**Figure 1:**
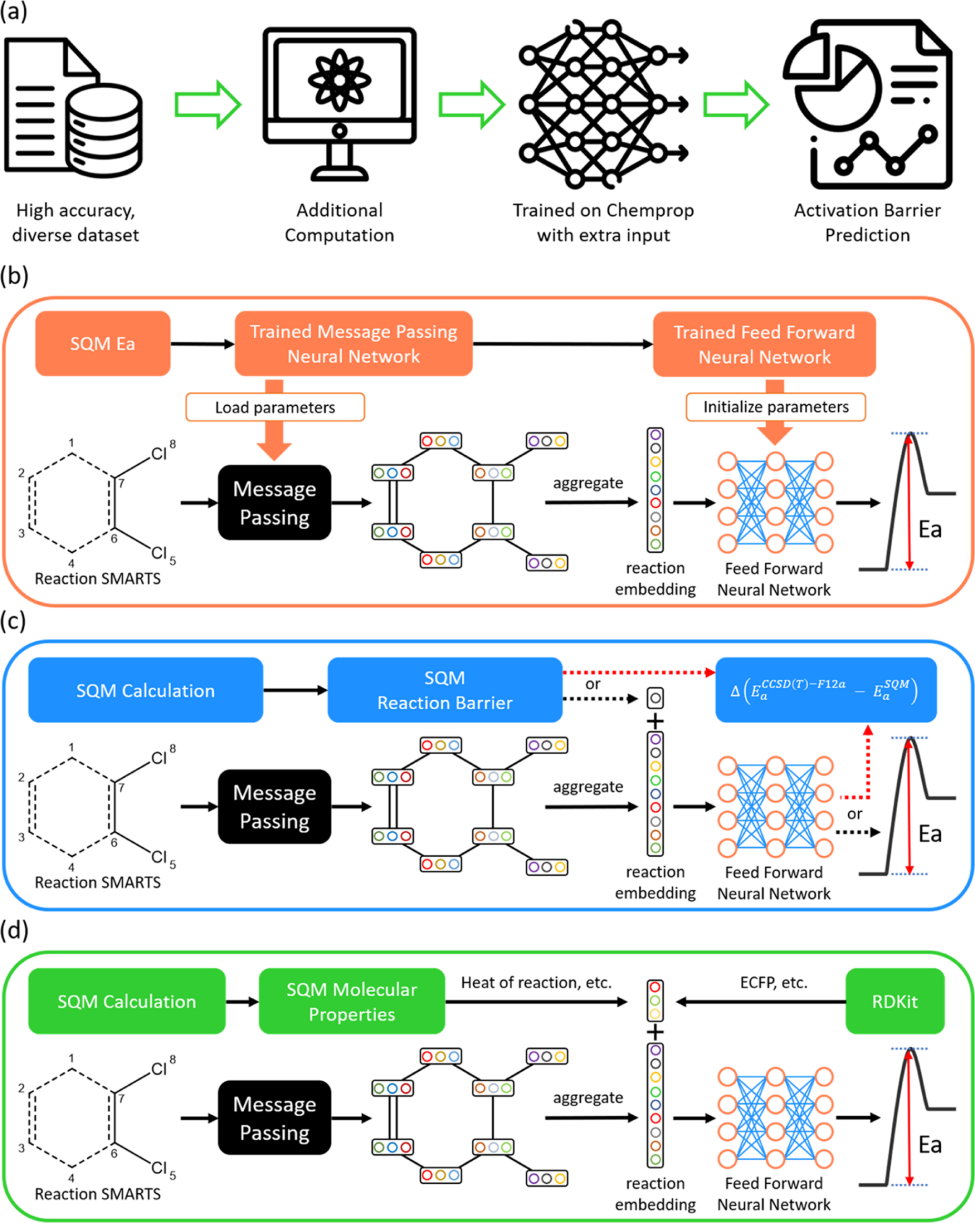
(a) Overview of the workflow: high-level activation energy data computed using CCSD(T)-F12a were collected as the reference data set. To explore data augmentation strategies, SQM calculations were performed to generate low-level data, which were integrated into GNN models to improve the activation energy predictions. Three data augmentation approaches were tested: (b) transfer learning approach, where the model is pretrained on low-level SQM activation energies and fine-tuned with high-level CCSD(T)-F12a data; (c) delta learning approach, which either uses low-level activation energy as an additional feature or predicts the difference between low- and high-level activation energies; and (d) feature engineering approach, incorporating properties such as the heat of reaction, calculated from low-level data, as additional input features for the model.

Building on these prior studies, our research aims to systematically investigate and compare the effectiveness of feature engineering, transfer learning, and delta learning applied to GNNs in predicting high-level CCSD(T)-F12a activation energies. These predictions are enhanced by augmenting data sets with low-cost, low-level SQM calculations, as illustrated in Figure 1. Our objective of this work is to identify the most relevant properties to compute and the optimal methods for integrating these into the model. For feature engineering, we focus on identifying features that significantly enhance the prediction of activation energies, conducting experiments on standardized benchmark data sets to evaluate the effectiveness of additional features compared to alternative methodologies. In transfer learning, we examine the impact of different pretraining domains and data quantities on model performance, comparing results with other methods to assess transfer learning’s efficacy in our specific context. Additionally, we evaluated the performance of delta learning within our benchmark framework, aiming to provide a comprehensive comparison of these three approaches in enhancing model accuracy across various reaction types.

The significance of this work lies in its potential to bridge the gap between the current capabilities of GNN models and the practical requirements of chemical reaction engineering. By rigorously evaluating and optimizing these advanced methodologies, our study not only contributes to the theoretical understanding of kinetic parameter prediction but also offers practical guidelines for developing more accurate and efficient models. These advancements could accelerate the discovery and design of new materials and chemical processes, thereby having a profound effect on various scientific and industrial applications.

## 2 Method

### 2.1 GNN Model Architecture

**Figure 2:**
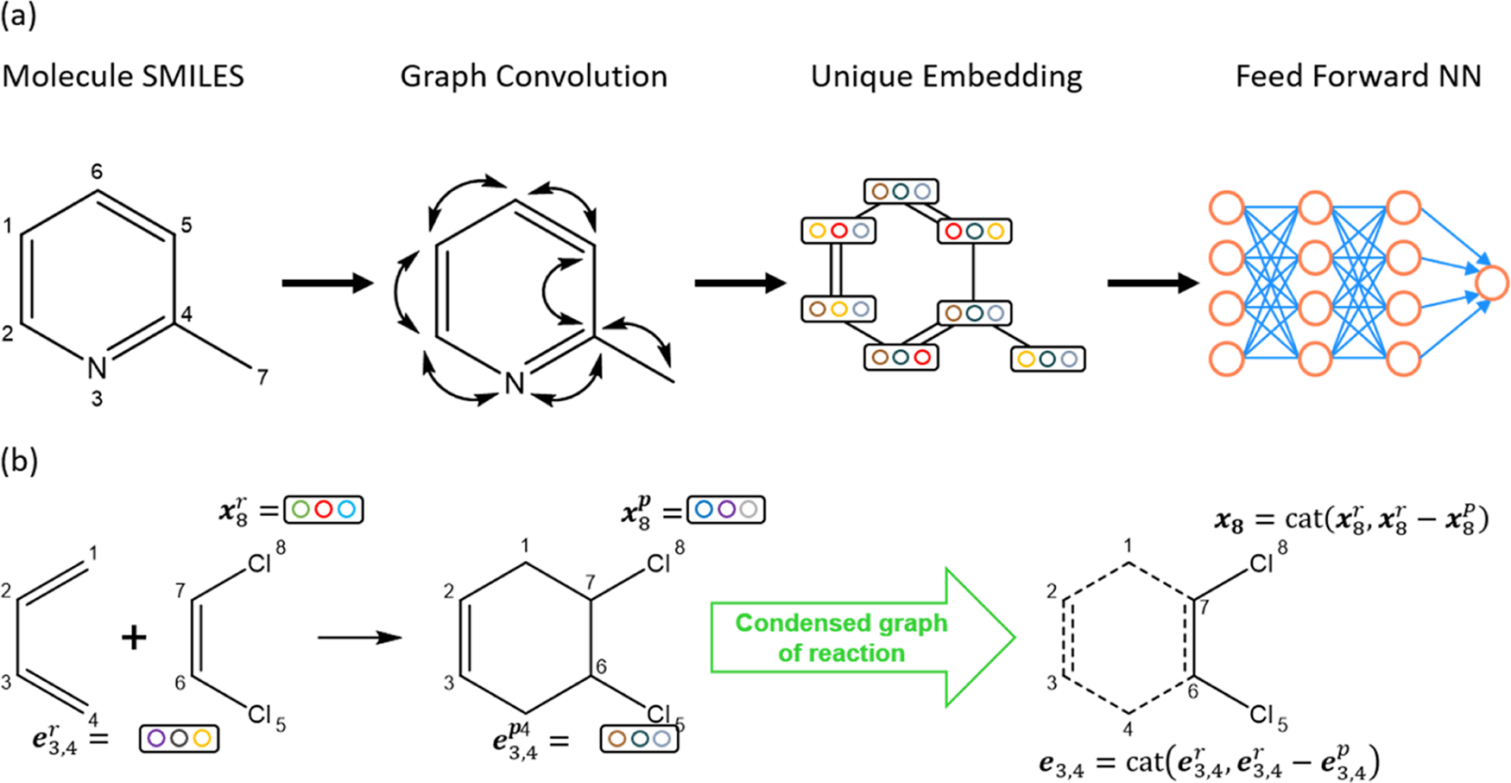
(a) Schematic of the Chemprop architecture. (b) Example of a condensed graph of reaction representation using the “reac_diff” mode. In the schematic, “**x**” denotes nodes, “**e**” represents edges in the GNN, and “r” and “p” correspond to reactant(s) and product(s), respectively.

In this work, we utilize Chemprop,^63^ a state-of-the-art GNN model, for the prediction of activation energies. Chemprop processes molecular structures by using SMILES strings to generate unique molecular descriptors. The model employs a D-MPNN, a type of GNN, to construct these descriptors. These descriptors, represented as vectors, are subsequently passed through a feed-forward neural network (FFNN) for target property prediction as shown in Figure 2a. Chemprop begins by describing molecules based on their fundamental properties and atomic connections. The atomic features include one-hot encodings of the atomic number, number of bonds, formal charge, chirality, number of hydrogens, hybridization, aromaticity, and scaled atomic mass. Bond features, such as bond type, bond conjugation, ring structure, and stereochemical information (e.g., cis/trans configuration), are also incorporated. This information is stored within the atom and bond features, which serve as the vertices and edges of the graph neural network. These features are iteratively updated through a message passing with neighboring atoms, creating an informative representation of each atom. After this exchange of information, the features are aggregated to form a comprehensive molecular representation.

To extend the D-MPNN architecture for reaction property prediction, Chemprop incorporates the CGR method,^19^ which enables users to input SMARTS,^64^ an efficient notation for concatenating the SMILES of reactants, solvents, and products. CGR represents chemical reactions by combining the reactant(s) and product(s) into a single molecular graph. A key advantage of CGR is its ability to connect disjoint parts between reactant and product graphs, facilitating the passing of messages among all atoms. As a result, the model architecture remains consistent and can be directly applied to the reaction tasks. Chemprop offers three modes for utilizing CGR with input reaction SMILES: “reac_diff,” “prod_diff,” and “reac_prod.” These modes correspond to (1) the reactants plus the difference between the product and reactants, (2) the product plus the difference between the reactants and product, and (3) the direct joining of reactants and product, respectively. Previous studies by Heid and Green have reported that the “reac_diff” mode (reactant + difference) generally provides the best performance.^65,66^ Therefore, in the following analyses, we use the CGR in its default mode (“reac_diff”), as shown in Figure 2b.

### 2.2 High-Level Data Set

To evaluate the performance of different data augmentation approaches, we selected a diverse and high-quality data set of reaction activation energies published by Spiekermann et al.^67^ This data set originates from the work of Grambow et al., where reactions were calculated at the ωB97X-D3/def2-TZVP level of theory.^68^ The molecules involved in these reactions were selected from the GBD-7 database and are composed of H, C, N, and O atoms with up to seven heavy atoms per molecule. The atom-mapped SMILES for these reactions were cleaned and updated using RDKit.^66^ In the study by Spiekermann et al.,^67^ these reactions were reoptimized at the ωB97X-D3/def2-TZVP level, with single-point energies refined at the CCSD(T)-F12a/cc-pVDZ-F12 level of theory. The original CCSD(T)-F12a data set comprises 11,927 reactions. However, since both transfer learning and delta learning require low-level SQM activation energies, as discussed below, we excluded reactions that failed to converge using the low-level methods. After removing these nonconverging reactions, the final high-level CCSD(T)-F12a data set used in this work consisted of 11,862 reactions. For the preparation of our training, validation, and testing data sets, we employed an 80:10:10 scaffold split on the CCSD(T)-F12a data set based on the SMILES strings. This partitioning method utilizes the Bemis–Murcko scaffold,^69^ as scaffold splits provide a more robust assessment of model generalizability compared to random splits.^65,70,71^

### 2.3 Enhancing ML with Low-Level Computational Data

In this study, we systematically explored various approaches for utilizing low-level computational data to enhance the performance of GNN models in predicting activation energies. These approaches differ in both the types of properties calculated using low-level methods and the ways in which these data are integrated into the model during training and prediction. To minimize computational costs associated with data generation, we focused on SQM methods, which are significantly more efficient than higher-level methods like DFT. Among the SQM methods we examined, AM1, PM3, and GFN2-xTB, we found that GFN2-xTB provided the lowest error in activation energy calculations. Consequently, GFN2-xTB was selected to generate the low-level data used in this study. The primary objective was to determine the most effective way to leverage these low-cost, low-level calculations to improve model performance, particularly when the availability of high-level data is limited.

The first approach that we investigated was transfer learning. As illustrated in Figure 1b, this method involves initially training the D-MPNN model on a large data set of activation energies computed with SQM methods. This pretraining phase allows the model to learn fundamental relationships between chemical structures and their associated activation energies from a substantial volume of low-level data. After this pretraining, the model is then fine-tuned using high-level CCSD(T)-F12a data to improve its predictive accuracy.

The second approach that we examined was delta learning. As illustrated in Figure 1c, delta learning differs from transfer learning in that it directly uses low-level activation energy data to predict the target high-level activation energies. This can be achieved either by incorporating the low-level activation energy as an additional input feature in the FFNN or by setting the model to predict the difference between the low- and high-level activation energies. This approach allows the low-level activation energies to be corrected by the model’s predictions, resulting in more accurate high-level activation energy estimates.

The third approach we explored was feature engineering, as depicted in Figure 1d. Unlike transfer learning and delta learning, which focus on low-level activation energy data, feature engineering involves using low-level calculations to compute other properties that may be related to the activation energy. As detailed in Table 1, we considered various properties related to thermodynamics, electronic structure,^51^ and hard and soft acids and bases (HSAB).^72^ For thermodynamic properties, we calculated these for both reactants and products and then determined the changes occurring during the reaction by subtracting the reactant properties from the product properties. These differences were used as additional inputs to the model. For electronic and HSAB properties, we followed the approach of Marques et al.,^51^ computing the properties of the reactants and incorporating them as additional inputs to the model. Detailed calculation procedures are provided in the Supporting Information. These computed properties were then combined with the reaction representation generated by the GNN and subsequently fed into the FFNN model to improve the accuracy of activation energy predictions.

**Table 1:** Computed Properties Used for Feature Engineering in the Activation Energy Prediction

	feature description
thermodynamic properties	electronic energy + ZPE correction
	enthalpy
	entropy
	Gibbs free energy
electronic properties	highest occupied molecular orbital
	lowest unoccupied molecular orbital
	gap between HOMO and LUMO
hard and soft acids and bases (HSAB)	hardness
	softness
	chemical potential
	electrophilic reactivity

### 2.4 Computational Details

To generate low-level SQM activation energies, we optimized all reactions from the high-level data set using AM1, PM3, and GFN2-xTB methods. Reactions that did not converge successfully were excluded from further analysis. For each reaction, the zero-point energy was calculated by performing harmonic vibrational analysis and added to the electronic energies of the reactant, product, and transition state. To ensure accuracy, a scaling factor was applied to the computed harmonic frequencies when calculating the zero-point vibrational energy. The scaling factors used were 0.948 for AM1, 0.940 for PM3,^73^ and 0.990 for GFN2-xTB.^74^ Additionally, the extra features utilized in our study, as listed in Table 1, were calculated using GFN2-xTB. All SQM calculations were conducted using the ORCA software package.^75^ For the Chemprop predictions, we employed an ensemble approach, using five models trained with different initial weights to minimize the model bias.

## 3 Results and Discussion

### 3.1 Baseline Model Development and Performance Evaluation

Before conducting comparisons, we first established a baseline model by training Chemprop exclusively on the high-quality CCSD(T)-F12a data set. The model was trained using both the default hyperparameters provided by Chemprop and after performing hyperparameter optimization (detailed in Tables S1 and S2 in the Supporting Information). The performance of the model on the test set is presented in Figure S3. Using the default parameters, the Chemprop model achieved a mean absolute error (MAE) of 6.98 kcal/mol and a root-mean-square error (RMSE) of 9.74 kcal/mol in predicting activation energies, indicating a significant prediction error (Table 2). After hyperparameter optimization, the MAE and RMSE were reduced slightly to 6.16 and 8.86 kcal/mol, respectively. A detailed comparison with other GNN models is available in the Supporting Information (Table S6).

**Table 2:** Reference Model Performance

hyperparameters	MAE (kcal/mol)	RMSE (kcal/mol)	*R*^2^ (−)
default	6.98	9.74	0.786
optimized	6.16	8.86	0.823

These results suggest that, despite Chemprop’s proven success in predicting a wide range of molecular properties, it faces challenges in accurately predicting activation energies in our study. This suboptimal performance may be attributed to the limited amount of training data, which appear insufficient for effectively training the model, particularly when applied to a scaffold-split test set. In this context, the test set comprises molecular structures not encountered during training, making these predictions particularly challenging for the model. While increasing the amount of CCSD(T)-F12a training data could potentially improve model performance—an approach supported by the work of Spiekermann et al.,^53^ who demonstrated that incorporating activation energies of reverse reactions can enhance model accuracy—we chose not to expand the high-level data in this study. The primary goal of this work is to investigate the effectiveness of different approaches for incorporating low-level data. Therefore, we maintained a constant amount of high-level data without additional augmentation and focused on evaluating the efficacy of various low-level data integration strategies using the Chemprop model with the optimized hyperparameter set (Table S1).

### 3.2 Evaluation of SQM Methods for Low-Level Calculations

**Figure 3:**
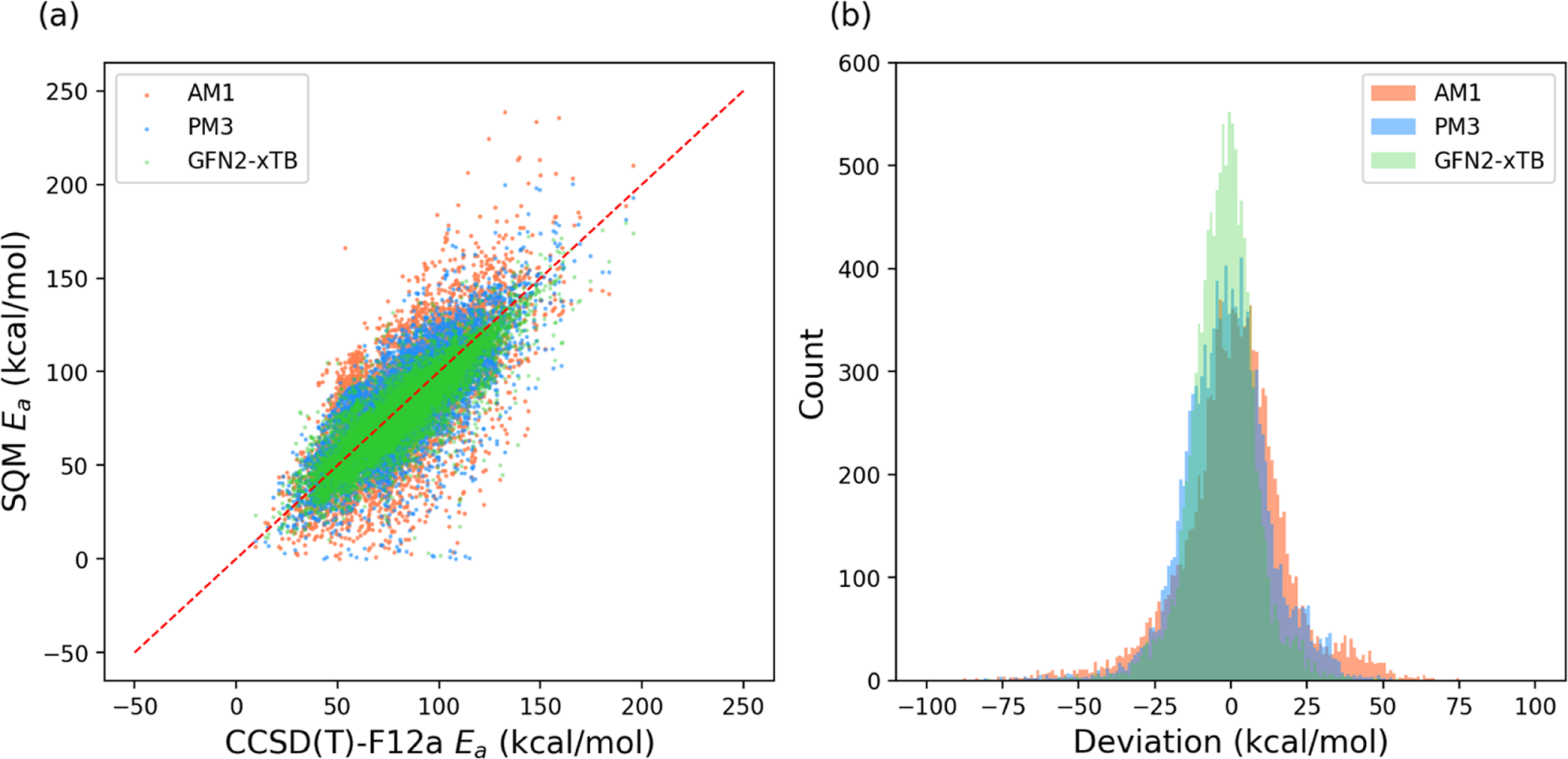
(a) Parity plots and (b) error distributions of activation energies calculated using AM1, PM3, and GFN2-xTB compared to high-level CCSD(T)-F12a reference data.

To select the most appropriate method for generating low-level data, we evaluated three SQM methods, AM1, PM3, and GFN2-xTB. We computed activation energies using these methods and compared them to the high-level CCSD(T)-F12a activation energies. As shown in Figure 3, the error distributions for all three SQM methods are centered around zero, indicating the absence of significant systematic errors. However, the MAEs for AM1, PM3, and GFN2-xTB were 13.02, 10.87, and 8.08 kcal/mol, respectively, indicating a broad error distribution and suggesting that activation energies calculated with these SQM methods may be associated with considerable error (Table 3). Despite this, GFN2-xTB demonstrated the lowest MAE, which is consistent with its reputation for having robust parametrization.^76^ Based on these findings, GFN2-xTB was selected to generate the low-level data used in subsequent analyses as it provides the most accurate results among the methods evaluated. Further discussion on how the accuracy of SQM data influences the final model performance is available in the Supporting Information (Table S11).

**Table 3:** Statistical Errors of Activation Energies Calculated by Using Different SQM Methods

	MAE (kcal/mol)	RMSE (kcal/mol)	*R*^2^ (−)
AM1	13.02	18.05	0.314
PM3	10.87	14.67	0.546
GFN2-xTB	8.08	11.1	0.740

### 3.3 Transfer Learning

In this subsection, we explored the potential of using transfer learning to enhance the Chemprop model’s performance. This approach involves initially training the model on a large data set of activation energies computed with GFN2-xTB, followed by a second round of refining with the high-level CCSD(T)-F12a training data set to improve the model. The GFN2-xTB reactions were derived from the Reaction Graph Depth 1 (RGD1) data set,^77^ which contains approximately 168,000 organic reactions involving molecules with up to 10 heavy atoms. This low-level data set encompasses a broad range of chemical reactions, and analysis using RMG database template identified 16 distinct reaction types,^15^ as shown in Figure S1. Notably, a significant portion of the reactions in this data set do not match any RMG template, indicating that many reactions fall outside the scope of the RMG database.

Interestingly, as shown in Table 4, pretraining with the RGD1 reactions resulted in a slight decrease in the model performance on the test set. This finding suggests that while transfer learning has been effective in previous studies for leveraging large amounts of lower-quality data, it does not always guarantee an improvement in model accuracy. One possible explanation for the negative impact of pretraining with the RGD1 data set is the discrepancy between the pretrained and target domains—not only in the quality of data (GFN2-xTB vs CCSD(T)-F12a) but also in the reaction types covered by the data sets. As illustrated in Figures S1 and S2, the reaction distributions in the RGD1 data set differ significantly from those in the target domain (high-level CCSD(T)-F12a reactions). Consequently, pretraining with the RGD1 data set may have led to a set of weights that are more suited to the RGD1 reactions, but less applicable to the reactions in the target domain, even after fine-tuning with high-level data.

**Table 4:** Statistical Errors of Activation Energies Predicted by the Chemprop Model with and without Transfer Learning

	MAE (kcal/mol)	RMSE (kcal/mol)	*R*^2^ (−)
baseline (without pretraining)	6.16	8.86	0.823
pretrained with RGD1 reactions	6.26	9.01	0.816
pretrained with RGD1 and CCSD(T)-F12a reactions computed at GFN2-xTB	4.82	7.00	0.889

To mitigate this issue, we recalculated the CCSD(T)-F12a reactions using GFN2-xTB and included these data in the pretraining set alongside the RGD1 reactions. This approach allowed the model to encounter reactions from the target domain, albeit at a lower level of calculation, during the pretraining phase. This inclusion led to improved performance in predicting the activation energies of the CCSD(T)-F12a test set after fine-tuning (Table 4 and Figure S4). The results demonstrated a consistent improvement in both MAE and RMSE compared with the baseline model, indicating that the effectiveness of the transfer learning approach is significantly influenced by the similarity between the pretraining data set and the target task. Additionally, using reaction energy as the pretrained target (Table S5) yielded lower predictive performance than activation energy, underscoring the challenges of transferring between different targets.

### 3.4 Delta Learning

Similar to the approach used in transfer learning, delta learning seeks to leverage the activation energies computed by using GFN2-xTB to improve model performance. However, unlike transfer learning, which utilizes GFN2-xTB activation energy data for model pretraining, delta learning directly incorporates the GFN2-xTB activation energy of a specific reaction to assist in predicting the corresponding CCSD(T)-F12a activation energy. This can be achieved in two ways: by including the GFN2-xTB activation energy as an additional input feature in the model or by setting the model to predict the difference between the CCSD(T)-F12a and GFN2-xTB activation energies. The latter approach treats this difference as a correction factor to adjust the GFN2-xTB activation energy, thereby estimating the CCSD(T)-F12a activation energy. The performance of these two approaches is summarized in Table 5 and Figure S5. Incorporating the GFN2-xTB activation energy as an additional input feature improves the model’s accuracy in predicting the CCSD(T)-F12a activation energy, reducing the MAE from 6.16 to 3.97 kcal/mol. The approach of predicting the difference between the CCSD(T)-F12a and GFN2-xTB activation energies as a correction proves to be slightly more effective, further decreasing the MAE to 3.85 kcal/mol.

**Table 5:** Statistical Errors of Activation Energies Predicted by the Chemprop Model with and without Delta Learning

	MAE (kcal/mol)	RMSE (kcal/mol)	*R*^2^ (−)
baseline (without delta learning)	6.16	8.86	0.823
delta learning with low-level activation energy input	3.97	5.95	0.920
delta learning predicting energy difference	3.85	6.54	0.903

The superior effectiveness of delta learning can be attributed to several key factors. First, this approach trains the model to specifically identify and correct errors inherent in GFN2-xTB calculations. By focusing on predicting the correction rather than directly predicting the activation energy, the learning task is simplified. These corrections tend to follow more consistent patterns, making them easier for the model to accurately learn. Furthermore, delta learning likely enhances generalization to unseen data, as the model is trained on residuals rather than the full energy values, which reduces the risk of overfitting—particularly when the data set is limited in size or diversity. This improved generalization is critical for achieving reliable predictions across a wide range of reaction types. Collectively, these factors indicate that delta learning is a highly effective approach for enhancing the accuracy of activation energy predictions.

### 3.5 Feature Engineering

Unlike transfer learning and delta learning, which focus on low-level activation energy data, feature engineering involves using low-level calculations to compute additional properties that may be related to the activation energy. These computed properties are then added as input features to enhance the reaction representation generated by the D-MPNN model. Among the various properties we considered (Table 1), thermodynamic properties had the most significant impact on reducing errors in activation energy predictions, as shown in Table 6 and Figure S6. This improvement can be attributed to the positive correlation between activation energy and reaction energy, as described by the Bell–Evans–Polanyi principle.^78,79^ By incorporation of thermodynamic features, the model is better equipped to capture the underlying relationships that influence activation energy, leading to more accurate predictions.

**Table 6:** Statistical Errors of Activation Energies Predicted by the Chemprop Model with and without Additional Input Features

	MAE (kcal/mol)	RMSE (kcal/mol)	*R*^2^ (−)
baseline (only D-MPNN features)	6.16	8.86	0.823
D-MPNN + thermodynamic properties	5.58	8.11	0.851
D-MPNN + electronic properties	6.19	8.91	0.820
D-MPNN + HSAB	6.11	8.91	0.823
D-MPNN + RDKit_2D	6.07	8.81	0.824
D-MPNN + Morgan (count)	6.70	9.47	0.797
D-MPNN + Morgan (bit)	6.45	9.23	0.808

In contrast, while electronic and HSAB properties are often used to explain chemical reactivity, their inclusion did not result in a significant enhancement in our experiments. This limited improvement aligns with the findings of recent work by Li et al.,^80^ which demonstrated that adding quantum mechanical features only aids the prediction of activation energies using the D-MPNN model when the training data set is extremely small (less than 2000 samples), a threshold smaller than the data set used in our study.

We also explored the potential of using input features that do not require additional physical simulations, such as Morgan fingerprints and RDKit_2D descriptors,^66,81^ to augment the reaction representation generated by the D-MPNN model. However, these approaches did not significantly improve the model performance (Table 6 and Figure S7). This lack of improvement may be due to the fact that these fingerprints are based on the structures of the reactants and products—information that is already encoded in the D-MPNN reaction representation. As a result, they do not effectively enhance the model’s ability to predict activation energies.

### 3.6 Comparison of Methods for Enhancing the Activation Energy Predictions

**Figure 4:**
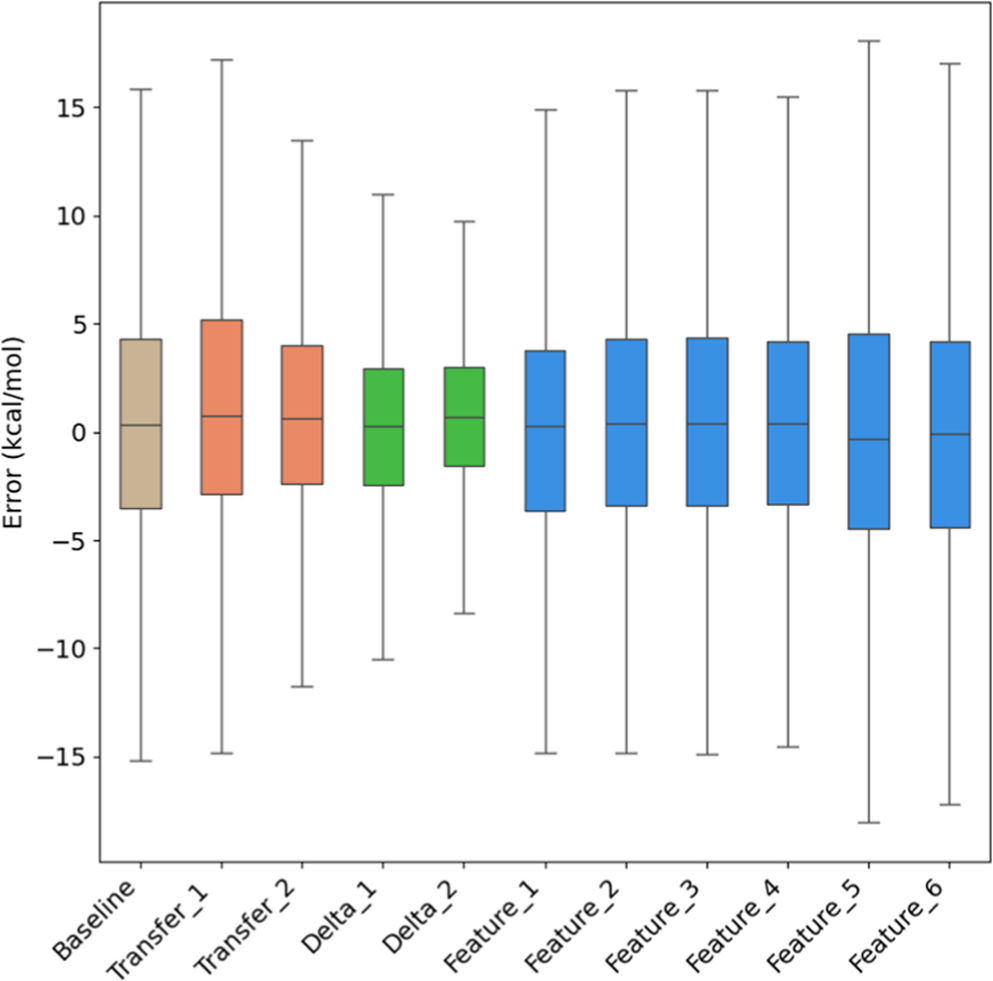
Error distribution of various data augmentation approaches for activation energy prediction: baseline model; transfer learning (Transfer_1: pretrained on RGD1 reactions; Transfer_2: pretrained on RGD1 and CCSD(T)-F12a reactions computed at the GFN2-xTB level); delta learning (Delta_1: using low-level activation energy as input; Delta_2: predicting the energy difference); and feature engineering (Feature_1: D-MPNN + thermodynamic properties; Feature_2: D-MPNN + electronic properties; Feature_3: D-MPNN + HSAB; Feature_4: D-MPNN + RDKit_2D; Feature_5: D-MPNN + Morgan (count); Feature_6: D-MPNN + Morgan (bit)).

To compare the performance of transfer learning, delta learning, and feature engineering, we analyzed the error distributions for each method, as shown in Figure 4. The delta learning approaches exhibit the narrowest error distributions compared to those of both the baseline model and the other data augmentation strategies. To further evaluate these methods, we selected the model with the lowest MAE from each method (best values in Tables 4–6) and summarized the results in Table 7. The results clearly indicate that delta learning is the most effective strategy for utilizing low-level data to enhance model accuracy in predicting activation energies. This trend remains robust even when alternative machine learning architectures were applied for activation energy prediction, as detailed in Table S8 of the Supporting Information. However, it is important to acknowledge that delta learning also imposes higher computational demands compared to other methods.

**Table 7:** Comparison of Statistical Errors in Activation Energy Predictions Using Chemprop with Transfer Learning, Delta Learning, and Feature Engineering

	transition state calculation required	additional computation at query	MAE (kcal/mol)	RMSE (kcal/mol)	*R*^2^ (−)
baseline	no	no	6.16	8.86	0.823
transfer learning	yes	no	4.82	7.00	0.889
delta learning	yes	yes	3.85	6.54	0.920
feature engineering	no	yes	5.58	8.11	0.851

Delta learning requires the calculation of low-level activation energies, which involves the challenging task of locating transition state structures. This process is significantly more complex than computing molecular properties for feature engineering, as transition state searches are nontrivial and often have a certain failure rate.^82^ This added complexity is analyzed in detail in Figure S9, which outlines the computational costs associated with each step of the molecular property calculations and transition state searches. Moreover, unlike transfer learning, which requires generating low-level training data only during the training phase, delta learning necessitates performing transition state searches for every query of activation energy during model application. This dependence increases both the computational burden and the operational complexity, necessitating robust methods to handle potential failures in transition state searches.

**Figure 5:**
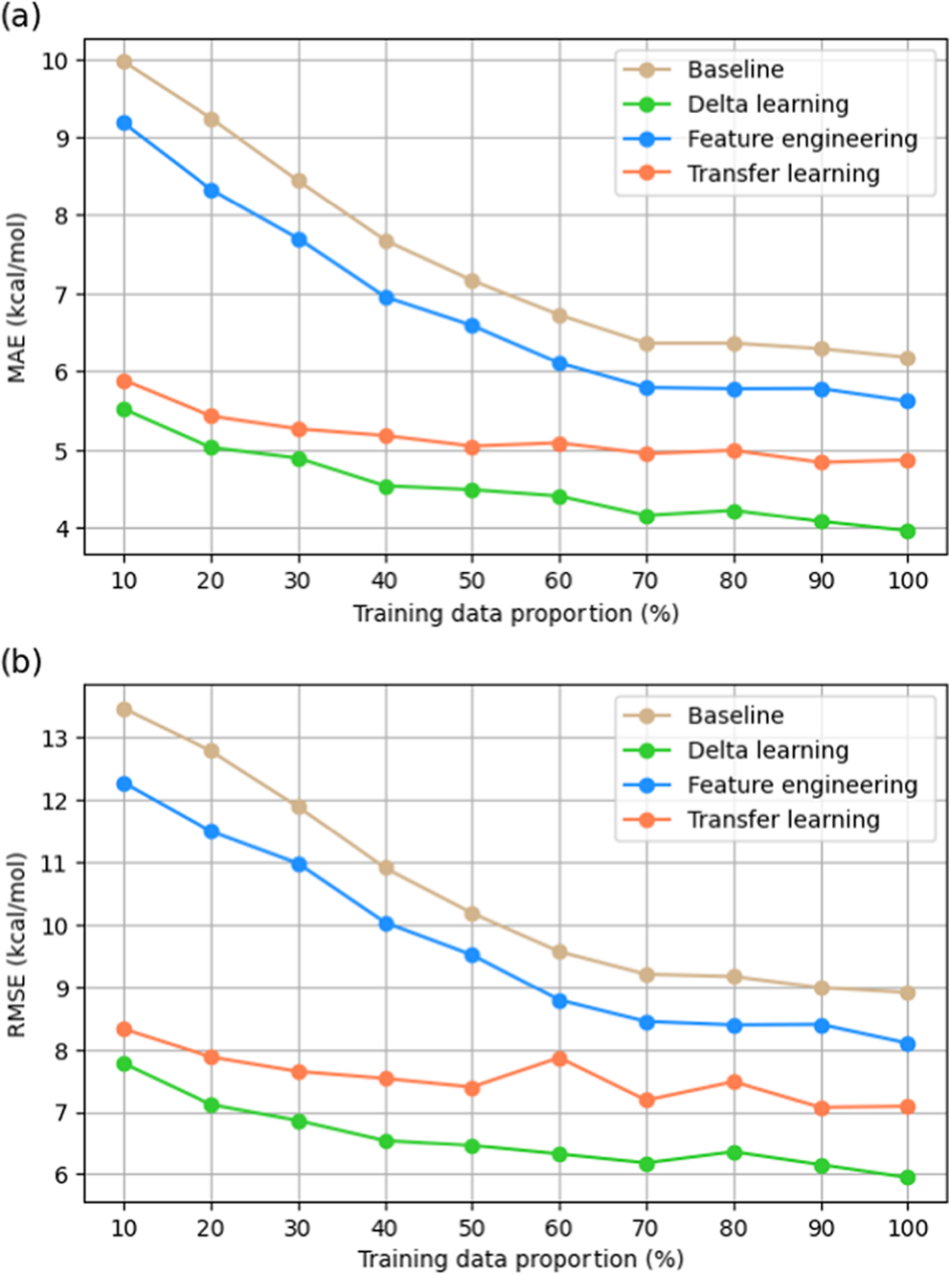
Performance comparison of delta learning, transfer learning, and feature engineering as a function of high-level training data availability. Models were trained on varying fractions (10–100%) of the CCSD(T)-F12a data set. With just 10% of the data, delta learning outperforms both the baseline model and feature engineering, even when they utilize the full data set. Additionally, delta learning achieves performance comparable to transfer learning trained on the full data set with only 20–30% of the data, highlighting its effectiveness in data-limited scenarios.

Despite its higher computational cost, delta learning offers distinct advantages. By using low-level activation energies for both training and querying, it simplifies the model’s task to learning corrections between low- and high-level activation energies. This focused approach reduces the dependence on high-level data, which are often expensive and time-consuming to produce. To assess the effectiveness of delta learning in reducing the demand for high-fidelity data, we evaluated model performance when trained on varying fractions (10–100%) of the original CCSD(T)-F12a data set. As shown in Figure 5, delta learning with just 10% of the high-level training data already outperforms the baseline model and feature engineering approaches that utilize the full data set. Additionally, delta learning trained with 20–30% of the high-level data achieves comparable performance to transfer learning trained with 100% of the high-level data. These findings indicate that delta learning is particularly advantageous in scenarios where high-level data availability is extremely limited, as it can achieve high accuracy with significantly less high-fidelity data despite requiring transition state searches at low-level theory during both training and querying.

In conclusion, delta learning delivers superior accuracy in activation energy predictions but comes with increased computational demands and operational challenges, particularly during low-level transition state calculations for both training and querying phases. The choice of the data augmentation strategy should be guided by the specific constraints and goals of the study. For cases in which computational resources are limited or transition state searches are infeasible during application, transfer learning and feature engineering may serve as more practical alternatives. However, when accuracy is the highest priority or high-level data are scarce, delta learning remains the most effective approach, offering substantial performance gains even under data-limited conditions.

## 4 Conclusions

In this study, we systematically explored and compared the effectiveness of three advanced data augmentation strategies (transfer learning, delta learning, and feature engineering) in enhancing the prediction accuracy of activation energies using the D-MPNN model. Each method utilized low-level computational data generated by SQM methods, specifically GFN2-xTB, to improve the model performance when high-level data availability is limited.

Our results indicate that delta learning is the most effective approach for leveraging low-level data to enhance the accuracy of activation energy predictions. By directly incorporating low-level activation energies or predicting the difference between low- and high-level energies, delta learning demonstrated superior performance in reducing prediction errors and required substantially fewer high-level data to achieve competitive performance. Specifically, delta learning trained with only 20–30% of high-level data achieved accuracy comparable to, or exceeding, that of other data augmentation methods trained with the full data set, making it particularly advantageous in data-scarce scenarios. However, this method also imposes significant computational demands, particularly due to the need for transition state structure searches during model application, which are both complex and resource intensive.

Transfer learning showed potential in leveraging pretraining data sets but delivered variable results depending on the alignment between the pretraining and target data sets. It remains a practical option when computational resources are limited or transition state searches are infeasible during querying. Feature engineering, while the least computationally demanding, provided only modest improvements with thermodynamic properties showing the most promise among the additional features considered.

Ultimately, the choice of method should be guided by the specific requirements and constraints of the study. Delta learning is ideal for achieving high accuracy with minimal high-level data. However, when computational efficiency is a priority or transition state searches are not feasible during model application, transfer learning and feature engineering may offer more practical alternatives. This work contributes valuable insights into the application of machine learning for chemical reaction engineering, providing practical guidelines for selecting appropriate data augmentation strategies to achieve reliable and accurate predictions in complex reaction systems.

## Data Availability

The CCSD(T)-F12a reaction data set can be accessed on Zenodo at 10.5281/zenodo.6618262. The RGD1 reaction data set is available at https://engineering.purdue.edu/savoiegroup/data+code.html. The GNN model used in this work is accessible on GitHub at https://github.com/chemprop/chemprop (version 1.6.1).

## Supporting Information Available

Additional information as noted in the text, including optimized model hyperparameters, reaction distribution analyses using RMG templates, detailed model performance, reaction energy in transfer learning, comparisons of GNNs for activation energy prediction, data augmentation across model architectures, the impact of SQM methods on model performance, HSAB descriptor calculations, and computational costs for low-level data generation (PDF)
