# Supplementary material for: Enhancing Activation Energy Predictions under Data Constraints Using Graph Neural Networks
Source: J Chem Inf Model. 2025 Jan 25;65(3):1367–77. doi: 10.1021/acs.jcim.4c02319 (PMC11815826; doi:10.1021/acs.jcim.4c02319)
Supplement: Supplementary file 1 — ci4c02319_si_001.pdf [file ci4c02319_si_001.pdf]

Enhancing Activation Energy Predictions under Data Constraints Using Graph Neural Networks

Han-Chung Chang,[a] Ming-Hsuan Tsai,[a] Yi-Pei Li[a][b]*

[a] Department of Chemical Engineering, National Taiwan University, No. 1, Sec. 4, Roosevelt Road, Taipei 10617, Taiwan.

[b] Taiwan International Graduate Program on Sustainable Chemical Science and Technology (TIGP-SCST), No. 128, Sec. 2, Academia Road, Taipei, 11529, Taiwan.

***Corresponding author:** Yi-Pei Li: yipeili@ntu.edu.tw

Supporting Information

(1) Model Hyperparameters

Hyperparameter optimization was conducted using the 'basic' argument as defined in Chemprop,[1] with the results summarized in Table S1. Any hyperparameters not listed in Table S1 were maintained at their default values as specified in Chemprop, as listed in Table S2.

Table S1. Optimized hyperparameters for the Chemprop model.

Hyperparameter	Value
Depth	6
Hidden_size	2000
FFN_fidden_size	2000
FFN_num_layer	3
Drouput	0.075

Table S2. Default hyperparameters for the Chemprop model

Hyperparameter	Value
Activation	ReLU
Aggregation	mean
Batch_size	50
Epochs	30
Init_lr	0.0001
Max_lr	0.001
Final_lr	0.0001
Loss_function	mse

(2) Reaction Distribution Analysis using RMG Templates

To evaluate the distribution of reactions within the CCSD(T)-F12a database[2] and the RGD1 database,[3] we utilized the reaction templates implemented in the Reaction Mechanism Generator (RMG).[4] For the reactions that could be analyzed using the RMG templates, we identified 14 distinct reaction types in the CCSD(T)-F12a database (Table S3 and Figure S1) and 16 reaction types in the RGD1 dataset (Table S4 and Figure S2).

Table S3. Distribution of RMG Reaction Types in the CCSD(T)-F12a Dataset.

RMG_family	Counts
Ketoenol	686
Singlet_Carbene_Intra_Disproportionation	256
1,3_Insertion_ROR	195
Retroene	173
2+2_cycloaddition	60
1,2_Insertion_CO	39
Intra_2+2_cycloaddition_Cd	18
1,3_NH3_elimination	12
Intra_ene_reaction	12
1,2_Insertion_carbene	8
1+2_Cycloaddition	7
6_membered_central_C-C_shift	4
1,3_Insertion_CO2	3
Diels_alder_addition	3

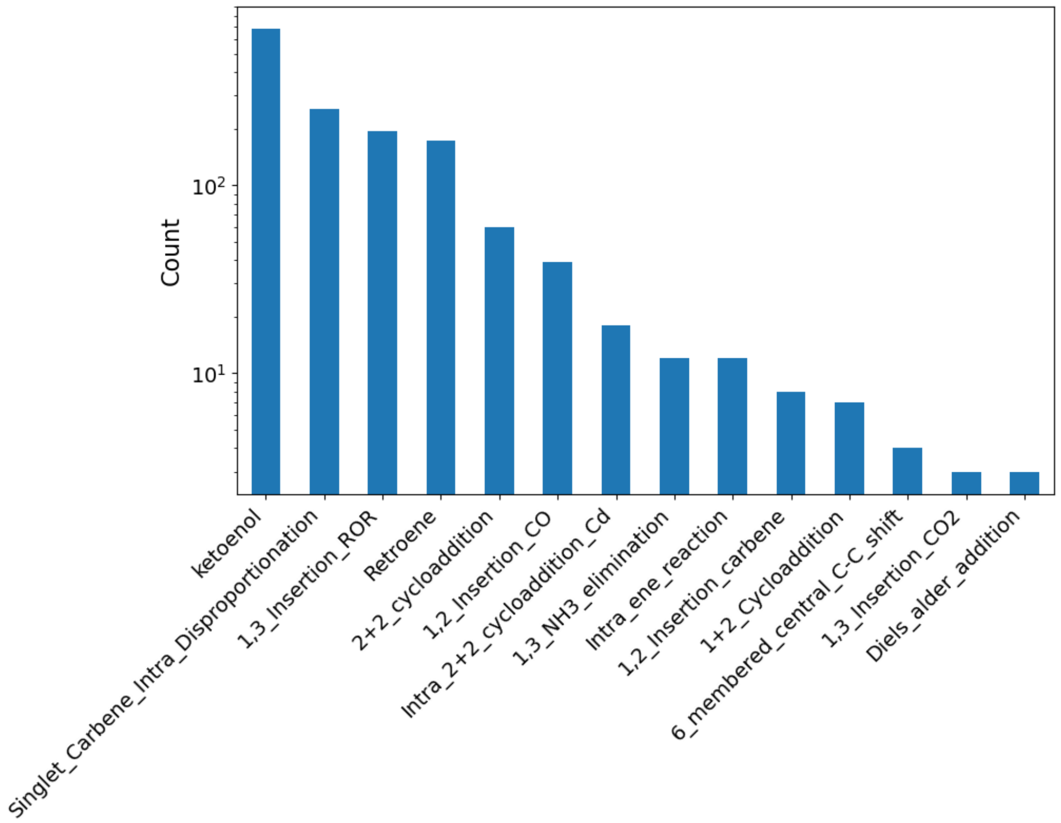

Figure S1. Histogram of RMG reaction types in the CCSD(T)-F12a dataset.

Table S4. Distribution of RMG Reaction Types in the RGD1 Dataset.

RMG_family	Counts
1,3_sigmatropic_rearrangement	12539
Retroene	1698
Intra_2+2_cycloaddition_Cd	1320
1,3_Insertion_ROR	1102
1,3_NH3_elimination	586
Ketoenol	291
1,3_Insertion_CO2	194
1,2_Insertion_CO	165
2+2_cycoaddition	156
Singlet_Carbene_Intra_Disproportionation	108
Diels_alder_addition	10
Intra_ene_reaction	8
Intra_Diels_alder_monocyclic	4
Intra_NO2_ONO_conversion	2
1,2_NH3_elimination	2
Cyclopentadiene_scission	1

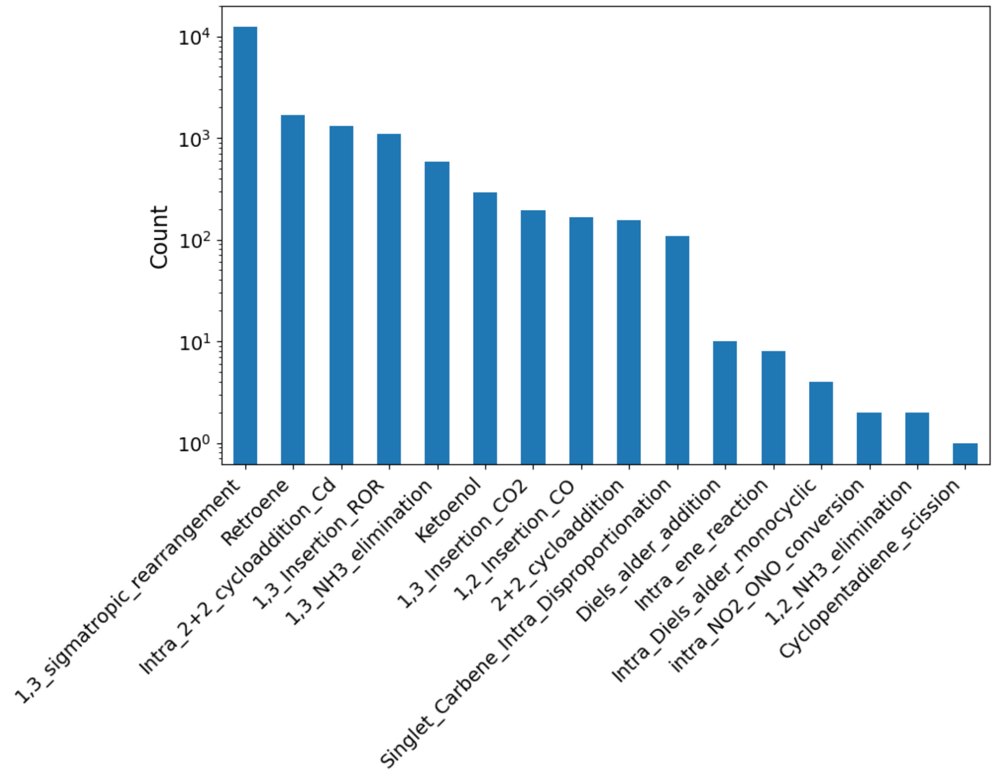

Figure S2. Histogram of RMG reaction types in the RGD1 dataset.

(3) Model Performance Details

The performance of the baseline model, transfer learning, delta learning, and feature engineering approaches on the CCSD(T)-F12a test set is illustrated through parity plots and deviation plots. The baseline model results, optimized with hyperparameter tuning, are shown in Figure S3. Figure S4 compares the baseline model with transfer learning models pretrained on RGD1 reactions and a combined RGD1/CCSD(T)-F12a dataset calculated with GFN2-xTB. Figure S5 examines delta learning models, highlighting predictions using low-level activation energy inputs and models trained to predict energy differences. Feature engineering approaches are detailed in Figures S6 and S7, where additional features such as D-MPNN descriptors combined with thermodynamic, electronic, and HSAB properties, as well as molecular fingerprints like RDKit_2D, Morgan bit, and Morgan count, are integrated into the models. Together, these figures present a comprehensive view of the model predictions, offering insights into their behavior and the influence of different training strategies and feature sets.

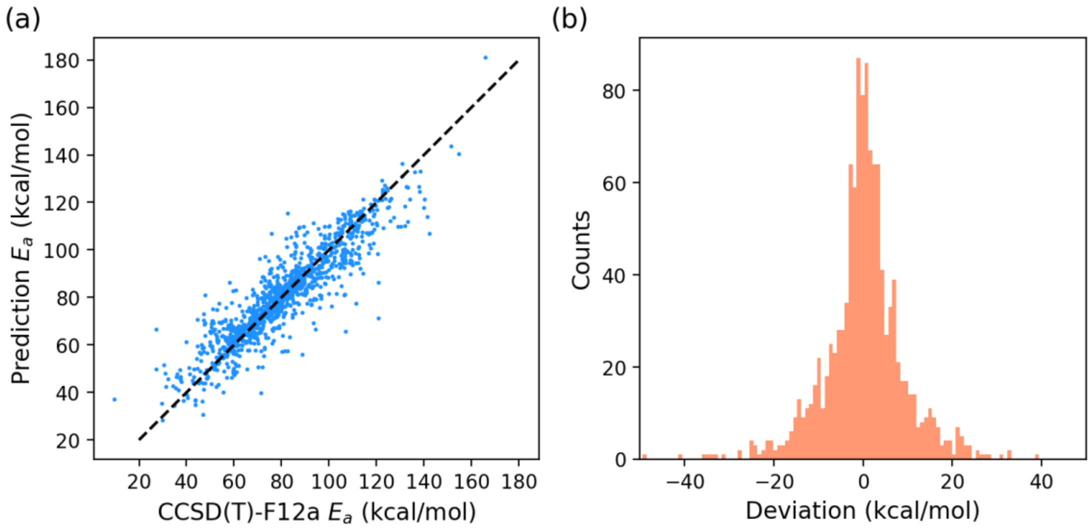

Figure S3. (a) Parity and (b) deviation plots of baseline model performance with the optimized hyperparameters.

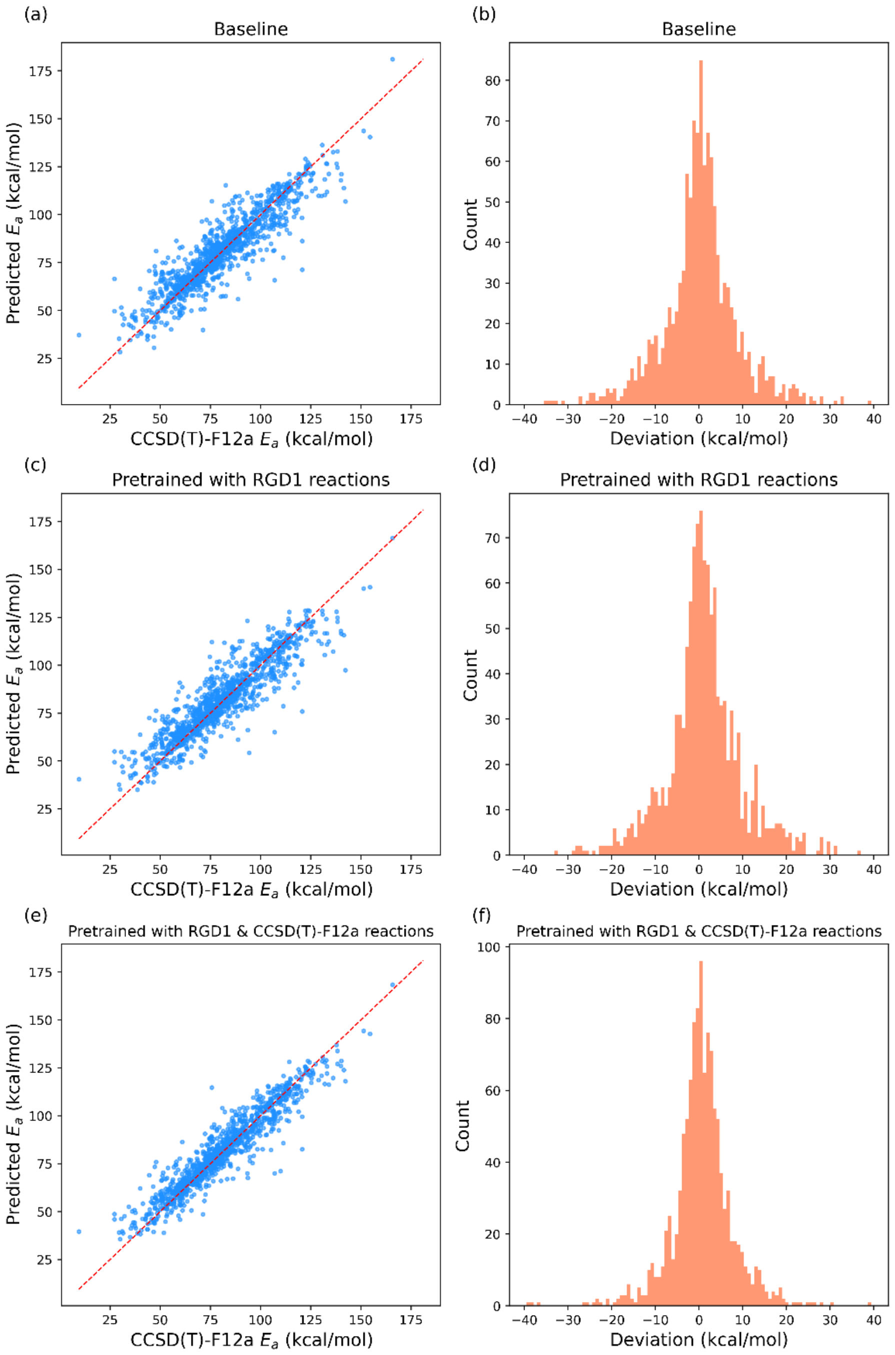

Figure S4. Parity plots and error distributions for activation energy predictions on the CCSD(T)-F12a test set using the baseline and transfer learning models: (a) and (b) show the baseline model, (c) and (d) present the model pretrained with RGD1 reactions, and (e) and (f) display the model pretrained with RGD1 and CCSD(T)-F12a reactions calculated using GFN2-xTB.

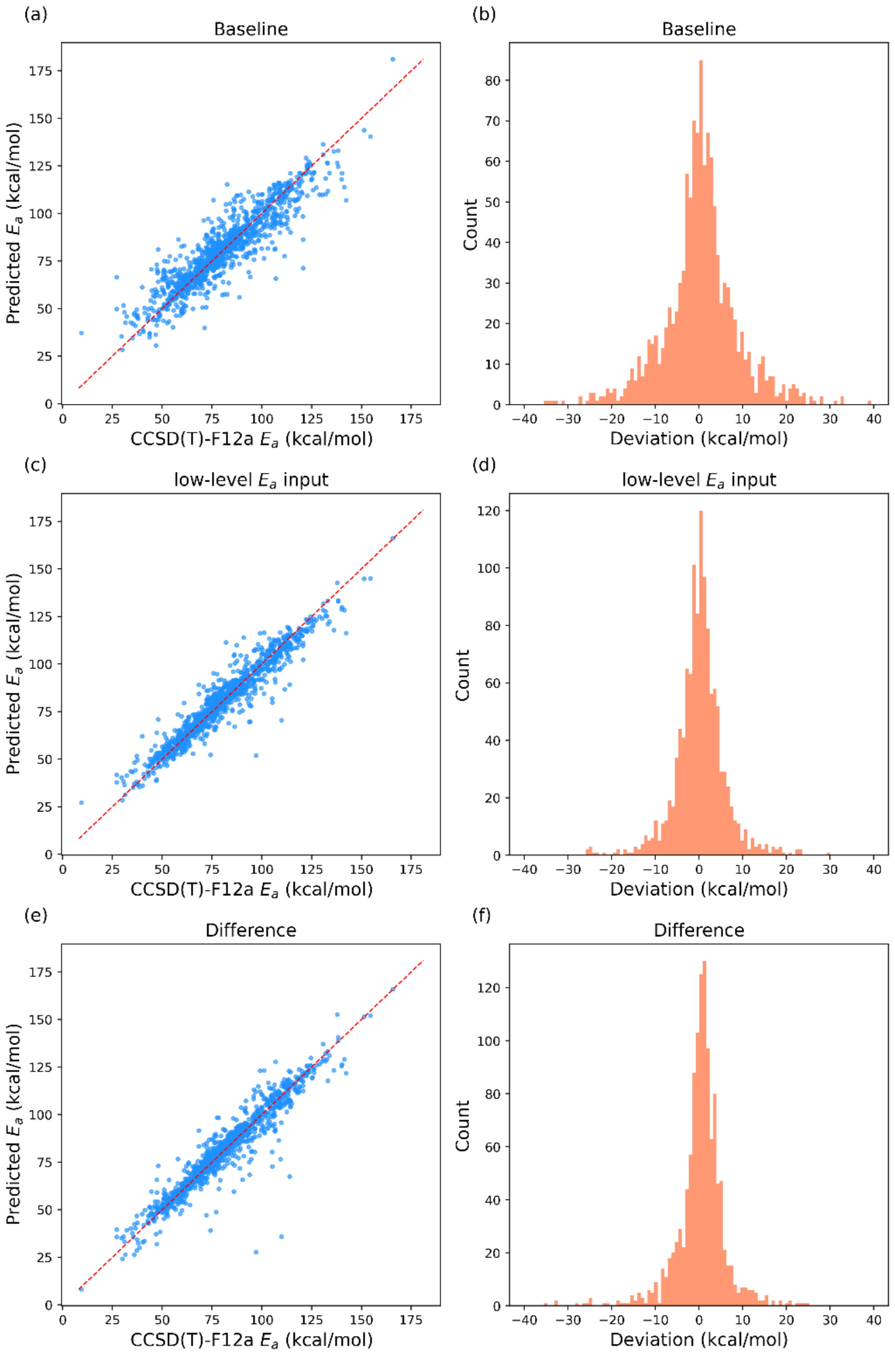

Figure S5. Parity plots and error distributions for activation energy predictions on the CCSD(T)-F12a test set using the baseline and delta learning models: (a) and (b) show the baseline model, (c) and (d) present the delta learning model with low-level activation energy input, and (e) and (f) display the delta learning model predicting the energy difference.

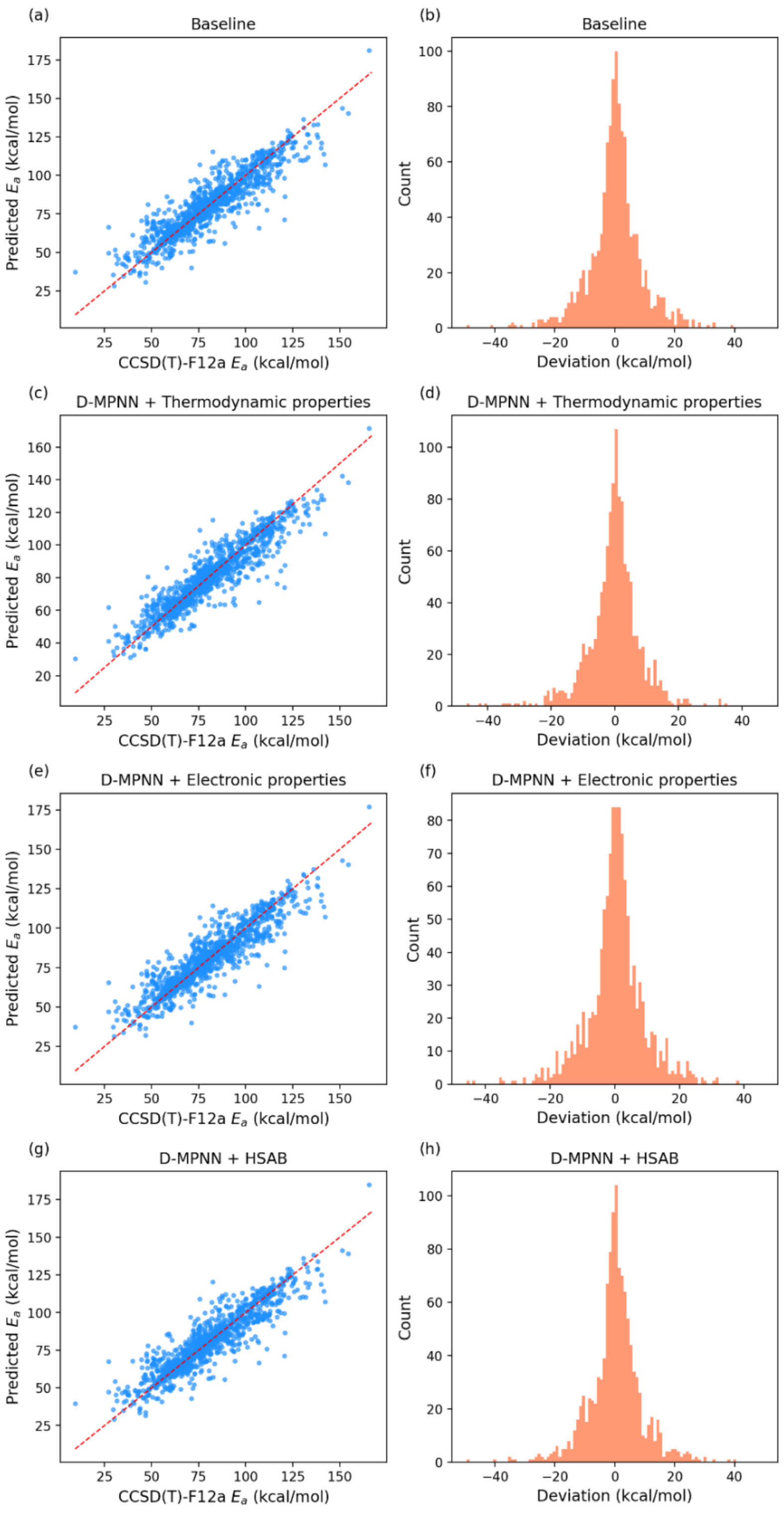

Figure S6. Parity plots and error distributions for activation energy predictions on the CCSD(T)-F12a test set using the baseline model and models with additional SQM computed features: (a) and (b) show the baseline model, (c) and (d) present the model with D-MPNN and thermodynamic properties, (e) and (f) display the model with D-MPNN and electronic properties, and (g) and (h) show the model with D-MPNN and HSAB properties.

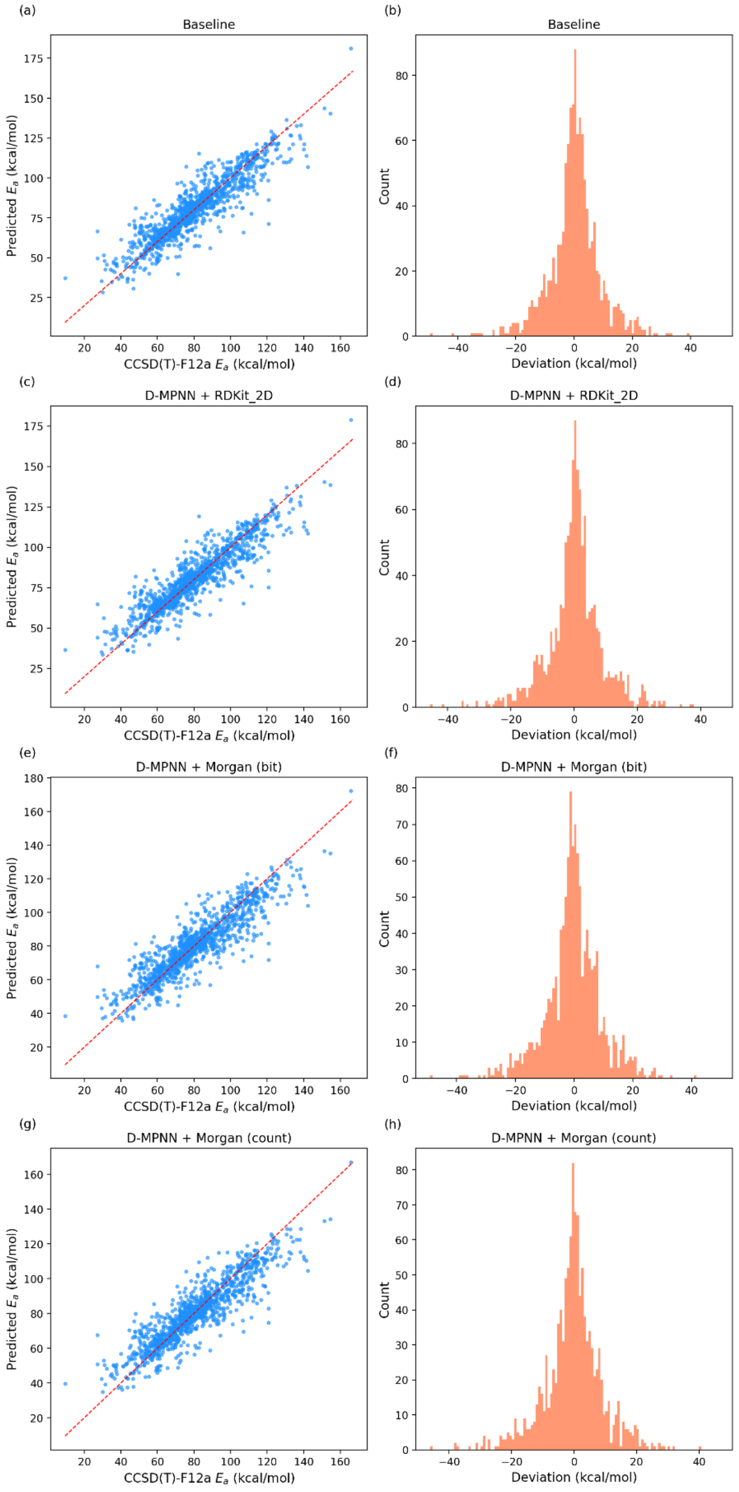

Figure S7. Parity plots and error distributions for activation energy predictions on the CCSD(T)-F12a test set using the baseline model and models with additional features: (a) and (b) show the baseline model, (c) and (d) present the model with D-MPNN and RDKit_2D fingerprints, (e) and (f) display the model with D-MPNN and Morgan bit fingerprints, and (g) and (h) show the model with D-MPNN and Morgan count fingerprints.

(4) Reaction Energy for Transfer Learning

Given the challenges in locating transition state structures, we explored the feasibility of using reaction energy as the pretrained target for transfer learning to bypass the need for transition state searches in low-level calculations. Specifically, we compared models pretrained using either low-level activation energies or reaction energies computed at the GFN2-xTB level of theory for reactions in the RGD1 + CCSD(T)-F12a datasets. The comparison, detailed in Table S5, shows that while using reaction energy as the pretrained target simplifies the workflow by avoiding transition state calculations, its predictive performance is lower than that of the model pretrained with low-level activation energies. These results suggest that transferring between different targets (e.g., reaction energy to activation energy) introduces additional complexity, which may limit the model's ability to generalize effectively.

Table S5. Performance Comparison of Transfer Learning Models Pretrained on Reaction Energy and Activation Energy.

Pretraining Target	MAE (kcal/mol)	RMSE (kcal/mol)	R^2 (-)
Low-level activation energy	4.82	7.00	0.889
Low-level reaction energy	6.30	9.18	0.810

(5) Comparison of GNNs for Activation Energy Prediction

In addition to the D-MPNN model implemented in Chemprop, we evaluated the performance of two additional GNN models: WL GNN and WL ml-QM-GNN.[5, 6] These models have demonstrated robustness in predicting specific reaction types, such as S_N2 and E2 reactions.[1] However, as shown in Table S6, they did not perform well on the dataset used in this work. A likely reason is that our dataset encompasses a diverse range of reactions, including cases where similar chemical transformations result in different activation energies. This complexity may pose challenges for the WL GNN and WL ml-QM-GNN models, as they primarily rely on reactant SMILES and reaction core as inputs. In contrast, the D-MPNN model in Chemprop incorporates the structures of both reactants and products, enabling it to better handle the variability and complexity present in this dataset. The hyperparameters for WL GNN models are documented in Table S7.

Table S6. Performance Comparison of Graph-Based Neural Network Models on the Target Reaction Dataset.

	MAE (kcal/mol)	RMSE (kcal/mol)
D-MPNN	6.16	8.86
WL GNN	26.9	33.6
WL ml-QM-GNN	26.5	33.2

Table S7. Hyperparameters for the WL (ml-QM-)GNN Model.

Hyperparameter	Value
depth	4
feature	50
hidden_size	128
max_nb	10
init_lr	0.001
selec_batch_size	10
selec_epochs	50
select_descriptors (ml-QM)	"partial_charge, fukui_elec, fukui_neu, nmr"
splits	[10, 10, 80]

(6) Data Augmentation Across Model Architectures

To evaluate the effectiveness of transfer learning, delta learning, and feature engineering across different model architectures, we applied the best-performing approach from each data augmentation strategy to a model using RXNFP—a chemical reaction fingerprint—as the encoder, combined with either FFNNs or XGBoost for activation energy prediction.[7, 8] The results, summarized in Table S8, align with the observations made using the D-MPNN model. Delta learning consistently outperforms both transfer learning and feature engineering across all model architectures. When comparing the overall performance of the different models, the D-MPNN model consistently outperforms RXNFP + FFNN and RXNFP + XGBoost, regardless of the data augmentation strategy applied. This is likely because the baseline D-MPNN model already demonstrates superior performance compared to the other two models, indicating its robustness and suitability for this task. The hyperparameters for RXNFP-based models are listed in Table S9 for FFNN and Table S10 for XGBoost.

Table S8. Comparison of Transfer Learning, Delta Learning, and Feature Engineering Across Different Models.

	D-MPNN			RXNFP + FFNN			RXNFP + XGBoost		
	MAE (kcal/mol)	RMSE (kcal/mol)	R^2 (-)	MAE (kcal/mol)	RMSE (kcal/mol)	R^2 (-)	MAE (kcal/mol)	RMSE (kcal/mol)	R^2 (-)
Baseline	6.16	8.86	0.823	12.7	17.3	0.327	12.6	17.1	0.338
Transfer learning	4.82	7.00	0.889	11.2	14.7	0.52	12.9	17.4	0.315
Delta learning	3.85	6.54	0.920	6.34	8.79	0.825	6.20	8.80	0.825
Feature engineering	5.58	8.11	0.851	12.2	16.2	0.406	11.7	15.9	0.430

Table S9. Hyperparameter Configuration for the RXNFP + FFNN Model.

Hyperparameter	Value
Depth	3
Hidden_size	2000
Learning_rate	0.001
Num_epochs	30
Batch_size	50

Table S10. Hyperparameter Configuration for the RXNFP + XGBoost Model.

Hyperparameter	Value
n_estimators	100
learning_rate	0.05
max_depth	6
subsample	0.8
colsample_bytree	0.8
random_state	42
early_stopping_rounds	15

(7) Impact of SQM Methods on Model Performance

To evaluate the impact of the accuracy of low-level data on the performance of the final model, we assessed model performance with delta learning (the most effective strategy for leveraging low-level data) using activation energies computed with AM1, PM3, and GFN2-xTB. The MAEs for activation energies from these methods were 13.02, 10.87, and 8.08 kcal/mol (Table 3), respectively, indicating varying levels of fidelity in the low-level calculations. The results reveal that the model's performance improves with increasing accuracy of the low-level data (Table S11), with GFN2-xTB yielding the best results, followed by PM3 and AM1. These findings highlight the importance of low-level method accuracy in determining the final model's predictive performance.

Table S11. Performance of Delta Learning with Different SQM Methods for Low-Level Data.

Low-Level Method	Prediction Type	MAE (kcal/mol)	RMSE (kcal/mol)	R^2 (-)
GFN2-xTB	Delta learning with low-level activation energy input	3.97	5.95	0.920
GFN2-xTB	Delta learning predicting energy difference	3.85	6.54	0.903
PM3	Delta learning with low-level activation energy input	5.14	7.13	0.885
PM3	Delta learning predicting energy difference	7.12	9.78	0.784
AM1	Delta learning with low-level activation energy input	5.17	7.35	0.878
AM1	Delta learning predicting energy difference	8.94	12.76	0.632

(8) Hard and Soft, Acid and Base (HSAB) Descriptors

This section details the calculation of additional HSAB descriptors, which provide insights into the electronic properties of reactants. According to previous work,[9] the formation of covalent bonds between reactant molecules can be understood through the characteristics of their outermost orbitals—specifically, the highest occupied molecular orbital (HOMO) and the lowest unoccupied molecular orbital (LUMO). Bond formation involves the transfer of electronic density between these orbitals. Marques et al.[10] further emphasize that incorporating electronic energy levels into reaction descriptions significantly enhances prediction accuracy and transferability.

The descriptors for hardness (η) and softness (σ) are defined as:

$$\eta = [E_{LUMO} - E_{HOMO}]/2 \tag{1}$$

$$\sigma = 1/\eta \tag{2}$$

where E_{LUMO} and E_{HOMO} represent the energies of the LUMO and HOMO, respectively. These values were extracted from frequency analysis performed at the GFN2-xTB level of theory. In addition to hardness and softness, we also calculated two related descriptors: chemical potential (μ) and electrophilic reactivity (ω), defined as:

$$\mu = [E_{LUMO} + E_{HOMO}]/2 \tag{3}$$

$$\omega = \mu^2/2\eta \tag{4}$$

These HSAB descriptors—hardness, softness, chemical potential, and electrophilic reactivity—were calculated and incorporated into the model as additional features. They were concatenated with the D-MPNN descriptors to enrich the input feature set and enhance model performance.

(9) Computational Costs of Low-Level Data Generation

In this section, we evaluate the computational costs associated with generating low-level data points for each data-augmentation scheme using the GFN2-xTB level of theory. The process involves several steps: geometry optimization of the reactant(s) and product(s), frequency analysis to confirm that the optimized structures correspond to local minima, and subsequent application of the climbing image nudged elastic band (CI-NEB)[11] method and transition state geometry optimization (TsOpt) to locate and refine the transition state structure. We analyzed the computational costs of these steps for 1,106 reactions extracted from the target dataset, with the cost distribution for each step illustrated in Figure S8. Among the steps, the CI-NEB procedure is the most computationally intensive, with costs approximately 1–2 orders of magnitude higher than the other steps. This is followed by transition state optimization, while geometry optimization and frequency analysis are the least demanding. For transfer learning and delta learning approaches, calculating the low-level activation energy necessitates performing both the CI-NEB and TS optimization steps, significantly increasing the computational burden. In contrast, the feature engineering approach requires only reactant and product geometry optimization and frequency analysis, resulting in considerably lower computational costs.

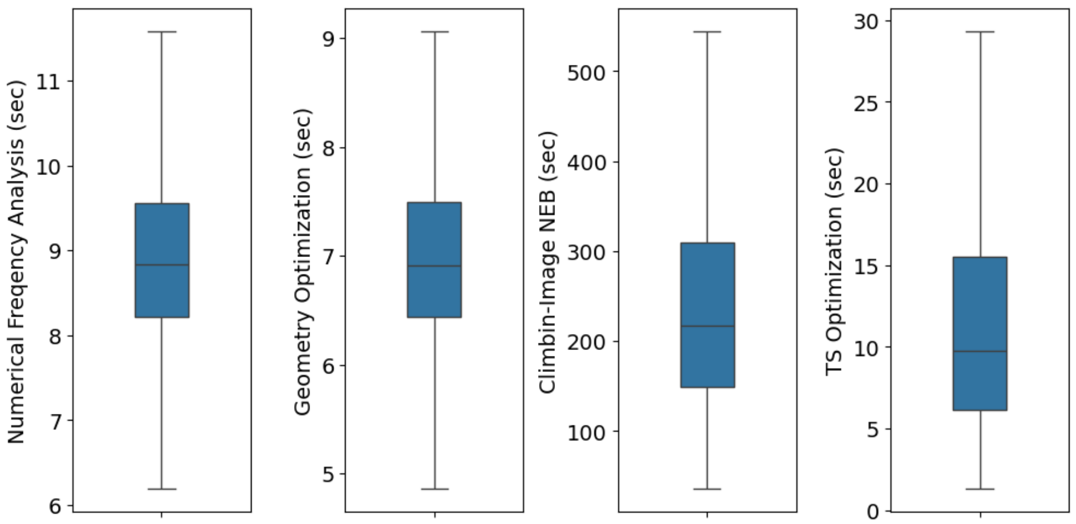

Figure S8. Box plot showing the computational time required for each step in generating a single data point. All calculations were conducted at the GFN2-xTB level of theory on AMD EPYC 7513 32-Core Processors. Geometry optimization and frequency analysis were parallelized across 32 cores, while CI-NEB and transition state optimization utilized 64-core parallelization.

Reference

(1) Heid, E.; Greenman, K. P.; Chung, Y.; Li, S.-C.; Graff, D. E.; Vermeire, F. H.; Wu, H.; Green, W. H.; McGill, C. J. Chemprop: A machine learning package for chemical property prediction. *Journal of Chemical Information and Modeling* **2023**, *64* (1), 9-17.
(2) Spiekermann, K.; Pattanaik, L.; Green, W. H. High accuracy barrier heights, enthalpies, and rate coefficients for chemical reactions. *Scientific Data* **2022**, *9* (1), 417.
(3) Zhao, Q.; Vaddadi, S. M.; Woulfe, M.; Ogunfowora, L. A.; Garimella, S. S.; Isayev, O.; Savoie, B. M. Comprehensive exploration of graphically defined reaction spaces. *Scientific Data* **2023**, *10* (1), 145.
(4) Liu, M.; Grinberg Dana, A.; Johnson, M. S.; Goldman, M. J.; Jocher, A.; Payne, A. M.; Grambow, C. A.; Han, K.; Yee, N. W.; Mazeau, E. J. Reaction mechanism generator v3. 0: advances in automatic mechanism generation. *Journal of Chemical Information and Modeling* **2021**, *61* (6), 2686-2696.
(5) Stuyver, T.; Coley, C. W. Quantum chemistry-augmented neural networks for reactivity prediction: Performance, generalizability, and explainability. *The Journal of Chemical Physics* **2022**, *156* (8).
(6) Stuyver, T.; Coley, C. W. Machine Learning-Guided Computational Screening of New Candidate Reactions with High Bioorthogonal Click Potential. *Chemistry–A European Journal* **2023**, *29* (28), e202300387.
(7) Schwaller, P.; Probst, D.; Vaucher, A. C.; Nair, V. H.; Kreutter, D.; Laino, T.; Reymond, J.-L. Mapping the space of chemical reactions using attention-based neural networks. *Nature machine intelligence* **2021**, *3* (2), 144-152.
(8) Chen, T.; Guestrin, C. Xgboost: A scalable tree boosting system. In *Proceedings of the 22nd acm sigkdd international conference on knowledge discovery and data mining*, 2016; pp 785-794.
(9) LoPachin, R. M.; Gavin, T.; DeCaprio, A.; Barber, D. S. Application of the hard and soft, acids and bases (HSAB) theory to toxicant–target interactions. *Chemical research in toxicology* **2012**, *25* (2), 239-251.
(10) Marques, E.; De Gendt, S.; Pourtois, G.; van Setten, M. J. Improving accuracy and transferability of machine learning chemical activation energies by adding electronic structure information. *Journal of Chemical Information and Modeling* **2023**, *63* (5), 1454-1461.
(11) Ásgeirsson, V.; Birgisson, B. O.; Bjornsson, R.; Becker, U.; Neese, F.; Riplinger, C.; Jónsson, H. Nudged elastic band method for molecular reactions using energy-weighted springs combined with eigenvector following. *Journal of chemical theory and computation* **2021**, *17* (8), 4929-4945.
